# Synovial membrane-derived mesenchymal progenitor cells from osteoarthritic joints in dogs possess lower chondrogenic-, and higher osteogenic capacity compared to normal joints

**DOI:** 10.1186/s13287-022-03144-z

**Published:** 2022-09-05

**Authors:** M. Teunissen, N. S. Ahrens, L. Snel, R. Narcisi, S. A. Kamali, G. J. V. M. van Osch, B. P. Meij, S. C. Mastbergen, K. Sivasubramaniyan, M. A. Tryfonidou

**Affiliations:** 1 Department of Clinical Sciences, Faculty of Veterinary Medicine, Utrecht University, Utrecht, The Netherlands; 2 Department of Orthopaedics and Sports Medicine, Erasmus MC, University Medical Center Rotterdam, Rotterdam, The Netherlands; 3 Galapagos Nederland, Leiden, The Netherlands; 4 Department of Otorhinolaryngology, Erasmus MC, University Medical Center Rotterdam, Rotterdam, The Netherlands; 5 Department of Rheumatology and Clinical Immunology, University Medical Center (UMC) Utrecht, Utrecht, The Netherlands

**Keywords:** Synovial membrane, Mesenchymal progenitor cells, Flow cytometry, Immunohistochemistry, Tri-lineage differentiation, CD271, CD34

## Abstract

**Background:**

Synovial membrane-derived mesenchymal progenitor cells (SM-MPCs) are a promising candidate for the cell-based treatment of osteoarthritis (OA) considering their in vitro and in vivo capacity for cartilage repair. However, the OA environment may adversely impact their regenerative capacity. There are no studies for canine (c)SM-MPCs that compare normal to OA SM-MPCs, even though dogs are considered a relevant animal model for OA. Therefore, this study compared cSM-MPCs from normal and OA synovial membrane tissue to elucidate the effect of the OA environment on MPC numbers, indicated by CD marker profile and colony-forming unit (CFU) capacity, and the impact of the OA niche on tri-lineage differentiation.

**Methods:**

Normal and OA synovial membrane were collected from the knee joints of healthy dogs and dogs with rupture of the cruciate ligaments. The synovium was assessed by histopathological OARSI scoring and by RT-qPCR for inflammation/synovitis-related markers. The presence of cSM-MPCs in the native tissue was further characterized with flow cytometry, RT-qPCR, and immunohistochemistry, using the MPC markers; CD90, CD73, CD44, CD271, and CD34. Furthermore, cells isolated upon enzymatic digestion were characterized by CFU capacity, and a population doublings assay. cSM-MPCs were selected based on plastic adherence, expanded to passage 2, and evaluated for the expression of MPC-related surface markers and tri-lineage differentiation capacity.

**Results:**

Synovial tissue collected from the OA joints had a significantly higher OARSI score compared to normal joints, and significantly upregulated inflammation/synovitis markers *S100A8/9*, *IL6*, *IL8*, and *CCL2*. Both normal and OA synovial membrane contained cells displaying MPC properties, including a fibroblast-like morphology, CFU capacity, and maintained MPC marker expression over time during expansion. However, OA cSM-MPCs were unable to differentiate towards the chondrogenic lineage and had low adipogenic capacity in contrast to normal cSM-MPCs, whereas they possessed a higher osteogenic capacity. Furthermore, the OA synovial membrane contained significantly lower percentages of CD90+, CD44+, CD34+, and CD271+ cells.

**Conclusions:**

The OA environment had adverse effects on the regenerative potential of cSM-MPCs, corroborated by decreased CFU, population doubling, and chondrogenic capacity compared to normal cSM-MPCs. OA cSM-MPCs may be a less optimal candidate for the cell-based treatment of OA than normal cSM-MPCs.

**Supplementary Information:**

The online version contains supplementary material available at 10.1186/s13287-022-03144-z.

## Background

Mesenchymal progenitor cells (MPCs), also referred to as mesenchymal stem/stromal cells (MSCs), are heterogeneous cell populations [1] residing in most adult tissues, including the synovial membrane [2, 3]. Transgenic animal models in combination with tissue injury models have demonstrated that synovial membrane-derived MPCs (SM-MPCs) contribute to joint development and tissue repair upon injury [4]. Therefore, SM-MPCs have been considered a promising cell-based treatment strategy for joint diseases such as osteoarthritis (OA) [5].

Under physiological conditions, MPCs participate in tissue homeostasis, remodelling, and repair by ensuring replacement of mature cells that are lost during physiological turnover, senescence, injury, or disease [6]. However, in the osteoarthritic joint, the delicate balance between anabolism and catabolism shifts towards a degenerative environment, characterized by the presence of inflammatory and catabolic mediators [7]. Due to these circumstances, and the poor healing capacity of the cartilage itself, the endogenous repair mechanisms become insufficient [8, 9]. This catabolic environment also influences the MPC populations in the joint. For example, bone marrow-derived MPCs from patients with hip OA possess a lower proliferation capacity and decreased chondrogenic differentiation capacity [10]. These findings, in addition to an increased senescence, were also reported for SM-MPCs from OA patients [11]. Furthermore, multiple studies show that the number of cells in the synovial membrane expressing MPC markers increases during OA progression [12–14] and their localization changes [12, 13]. However, as the exact origin, function and phenotype of MPCs in vivo remain elusive, additional research to elucidate the effect of OA on SM-MPCs and, in turn, their role during OA is necessary.

At this moment, the dog is considered a very relevant animal model for OA due to its translational values in terms of anatomic similarity, disease progression, and translation of outcomes to humans [15, 16]. Importantly, both natural occurring OA and surgically induced dog models exist [16, 17], and as such, treatment strategies can be evaluated early in product development for the target species [18]. The presence of SM-MPC has been demonstrated in the normal [19] and OA [20, 21] synovial membrane of dogs. However, a direct comparison between normal and OA canine SM-MPCs is lacking, hampering translational studies and advances in this field employing the dog as a model. In addition, the lack of canine-specific SM-MPC markers hampers the isolation and investigation of this cell population.

In this study, the characteristics of canine (c)SM-MPCs from normal and clinical OA joints were compared. cSM-MPCs of normal joints had higher chondrogenic and adipogenic capacity than cSM-MPCs from OA joints, while the latter showed a higher osteogenic capacity. Therefore, differences in the native cMPC population in the synovial membrane were investigated using flow cytometry, RT-qPCR and immunohistochemistry to investigate the expression profile of common MPC/MSC markers CD90, CD44, CD73, CD271, and CD34.

## Methods

### Terminology

In this manuscript, native, uncultured cells derived from the synovial membrane are referred to as mesenchymal progenitor cells (cSM-MPCs). Expanded cells, selected based on plastic adherence, are referred to as mesenchymal stromal cells (cSM-MSCs) (Additional file 1: Fig. S1).

### Animal Samples

Synovial membrane (SM) was collected from the inner side of the lateral and medial joint capsule of the clinically normal knee joints of skeletally mature, mixed-breed dogs (Additional file 1: Table S1, *n* = 29, 18 ± 7 months of age, 25 ± 2 kg), euthanized in unrelated experiments conducted in accordance with the guidelines set by the National Central Committee for Animal Experiments (AVD #115,002,016,531). SM from OA knee joints was collected from the inner side of the medial joint capsule, with the owner’s consent, during standard-of-care surgery (Additional file 1: Table S1; *n* = 22, 58 ± 37 months of age, 38 ± 12 kg) for cranial cruciate ligament rupture of client-owned dogs at the academic hospital of the Faculty of Veterinary Medicine of Utrecht University. All dogs suffered from secondary knee OA based on clinical examination with accompanying joint effusion, increase of intra-articular soft tissue density, and osteophyte formation as confirmed by radiography.

SM tissue samples were processed accordingly for histopathological examination, gene expression profiling, and cell culture experiments. For this purpose, SM was, respectively, (a) fixed in 4% neutral buffered formalin (NBF, Klinipath B.V., Duiven, The Netherlands) for paraffin-embedding and (immuno-) histochemical analysis, (b) snap frozen and stored at − 80 °C for RNA isolation, and (c) stored in αMEM (22561021, Gibco™, Thermo Fisher Scientific, Waltham, USA) supplemented with 1% ITS + Premix (354352, Corning Life Sciences, Amsterdam, The Netherlands), and 1% penicillin/streptomycin (p/s; 10.000 U/mL, 15140122, Gibco™) for a maximum of 24 h until tissue digestion. The use of tissue samples for specific outcome parameters was based on availability and was not stratified.

### Histopathological evaluation of the SM

To determine the OA status of the SM, 5 µm sections were stained with Haematoxylin/Eosin (HE) (Mayers haematoxylin (109249, Merck), 0.2% Eosin (115935, Merck)), randomized and scored blindly according to the Osteoarthritis Research Society International (OARSI) canine scoring system [16] by three observers (MT, LS, and SCM).

### Synovial membrane tissue digestion and isolation of cSM-MPCs from the synovial membrane

SM tissue samples were minced, and enzymatically digested using 2 mg/mL collagenase IV (C5138, Sigma-Aldrich) and 0.08 mg/mL dispase II (17105041, Gibco™) in Hanks’ Balanced Salt Solution (HBSS; 14025050, Gibco™), at 37 °C on a shaker for 2–3 h. Thereafter, remaining undigested tissue was removed by passing the digest through an 18G needle, and subsequently through 100 µm (542000, Greiner Bio-One, Alphen aan den Rijn, The Netherlands) and 40 µm (542040, Greiner Bio-One) cell strainers, respectively. The acquired cell suspensions were centrifuged for 8 min at 290*g* and washed twice with αMEM containing 10% foetal bovine serum (FBS; 16000044, Gibco™) and 1% p/s. The total and live cell numbers were determined with a TC20™ Automated Cell Counter (145-0101, Bio-Rad).

### Colony-forming unit capacity of cSM-MPCs

To evaluate the colony-forming unit (CFU) capacity, cSM-MPCs of normal (*n* = 8) and OA (*n* = 12) donors were plated in 58 cm^2^ Petri dishes (664160, CELLSTAR®, Greiner Bio-One) at three cell densities, i.e. ± 0.25-, 0.5-, and 1 × 10^3^ cells/Petri dish (equivalent to ± 4-, 8-, and 17 cells/cm^2^). After 10–14 days in humidified, normoxic conditions (5% CO_2_/21% O_2_) at 37 °C, normal culture conditions, cells were stained with 0.5% crystal violet (C0775, Sigma-Aldrich) in 100% methanol (MC1060092511, Merck Millipore) for 30 min. Colonies containing > 50 cells were counted in the appropriate plating density, i.e. the density wherein the individual colonies were not overlapping, and displayed as the percentage of the total seeded cell number.

### Expansion of cSM-MSCs

Following isolation, cells were plated at a density of ± 3.3 × 10^3^ cells/cm^2^ in T75 culture flasks (658175, CELLSTAR®, Greiner Bio-One), hereafter called cSM-MSCs. cSM-MSCs were cultured under normal culture conditions, in expansion medium (αMEM, 10% FBS, 1% p/s, 0.1 mM ascorbic acid 2-phosphate (AsAP; A8960, Sigma-Aldrich, Saint Louis, USA), 1.25 μg/mL Fungizone (Amphotericin B, 15290018, Gibco™) and 1 ng/mL recombinant human basic fibroblast growth factor-2 (bFGF-2; PHP105, Bio-Rad)). After ± 80% confluency was reached (normal: 6.9 ± 1.5 days; OA: 9.4 ± 2 days) in passage (P) P0, cSM-MSCs were aliquoted and cryopreserved in αMEM with 20% FBS and 20% dimethyl sulfoxide (102950, Merck Millipore, Burlington, USA) at − 196 °C until further analysis.

### Population doubling assay of cSM-MSCs until passage 10

Normal (*n* = 6) and OA (*n* = 6) cSM-MSCs were seeded in triplicate at a density of ± 6 × 10^3^ cells/cm^2^ in 6-well plates (657160, CELLSTAR®, Greiner Bio-one) under normal culture conditions in expansion medium. Cells were passaged every 3–4 days up to P10. Population doublings per passage was calculated with the following formula: PD = log(Nf) − log(Ni)/log(2), where PD stands for the number of cell divisions in each passage, Nf for the cell number on the day of passaging and Ni for the initial seeding number of cells (6 × 10^3^ cells/cm^2^).

### Senescence assay of P0 to P10 cSM-MSCs

As senescence is thought to play a significant role in OA, it was hypothesized that cSM-MSCs derived from OA joints might undergo senescence earlier compared to normal cSM-MSCs. Therefore, a senescence assay was performed in which normal (*n* = 6) and OA (*n* = 6) cSM-MSCs were seeded at a density of 2,5 × 10^4^ cells/cm^2^ in duplicate in chamber slides (PEZGS018, Millipore). After 24 h (37 °C, 5% CO_2_/21% O_2_), cells were fixed with 4% NBF and stained overnight at 37 °C in the dark with 5-bromo-4-chloro-3-indolyl-β-D-galactoside (X-gal (B4252, Sigma-Aldrich), 1 mg/ml, in a solution of 40 mM citric acid/sodium phosphate, 5 mM potassium hexacyano-ferrate (II) trihydrate, 5 mM potassium hexacyano-ferrate (III), 150 mM sodium chloride, and 2 mM magnesium chloride (MgCl_2_) in distilled water (pH 6.0)). To visualize nuclei, a counterstain with DAPI (62,248, ThermoFisher) was performed. Sections were mounted with FluorSave (345,789, VWR), and imaged using Olympus BX43 microscope (Olympus, Tokyo, Japan). Analysis of the percentage of senescent cells was performed with ImageJ (version 1.48) using the *Senescence Counter* [22], which uses the amount of DAPI positive nuclei to determine the amount of X-gal positive cells.

### Tri-lineage differentiation assay of P2 cSM-MSCs

Tri-lineage differentiation of the cSM-MSCs was performed in P2 as per methods described previously [23], with some small adaptations. For chondrogenic differentiation, normal (*n* = 7) and OA (*n* = 8) P2 cSM-MSCs were cultured in pellets of 100.000 cells/well in a 96-well plate (Corning® Costar® 7007) in chondrogenic differentiation medium (DMEM high glucose (31966, Invitrogen), 1% p/s, 1% ITS + Premix, 0.04 mg/mL proline (P5607, Sigma), 0.1 mM AsAP and 10^–7^ M dexamethasone (D1756, Sigma)) with the addition of 10 ng/mL recombinant human transforming growth factor-β1 (TGF-β1; 240-B, R&D Systems, Minneapolis, USA) and 100 ng/mL recombinant human bone morphogenetic protein-2 (BMP-2; 355-BM, R&D systems). After 21 days of culture (37 °C, 5% CO_2_/21% O_2_), pellets were collected for histological (*n* = 3/donor/condition) and biochemical (*n* = 3/donor/condition) evaluation. Histological evaluation of glycosaminoglycan (GAG) deposition using Toluidine blue O (0.04%, Sigma; Basic Blue 17, 86% dye, dissolved in 0.2 M acetate buffer), biochemical analysis of GAG release and deposition using a dimethyl methylene blue (DMMB, Sigma) assay, and determination of the DNA content using the Qubit™ dsDNA HS Assay (ThermoFisher Scientific), were performed as per methods described previously [23]. Considering that DNA content after 21 days of culture is a snapshot in time and does not represent the dynamics of cell numbers throughout culture, the GAG content of the pellet, the GAG excretion into the medium, and the DNA content were displayed separately.

For osteogenic and adipogenic differentiation, normal (*n* = 6) and OA (*n* = 6) P2 cSM-MSCs were seeded at a density of 1 × 10^3^ (osteogenic) or 4 × 10^3^ (adipogenic) cells/cm^2^ in 6-well plates. Technical duplicates per donor were cultured in osteogenic differentiation medium (DMEM with 10% FBS, 0.1 mM AsAP, 1.25 μg/mL Fungizone, 10 mM β-glycerophosphate (G9422, Sigma-Aldrich), and 10^–7^ mM dexamethasone) or adipogenic differentiation medium (DMEM with 10% FBS, 0.1 mM AsAP, 1.25 μg/mL Fungizone, 10^–6^ mM dexamethasone, 0.2 mM indomethacin (I7378, Sigma-Aldrich), 0.01 mg/mL recombinant human insulin (I9278, Sigma-Aldrich), 0.5 mM 3-isobutyl-1-methylxanthine (I5879, Sigma-Aldrich), and 5 µM rosiglitazone (R2408, Sigma-Aldrich)) for staining and for gene expression analysis. Negative controls received expansion medium. After 21 days, cell monolayers were fixed with 4% NBF, and stained with Alizarin Red S staining solution (2% Alizarin Red S (A5533, Sigma-Aldrich)) for evaluation of calcium deposits or Oil Red O staining solution (0.3% Oil Red O (O0625, Sigma-Aldrich)) for evaluation of intra-cellular lipid droplets. The Alizarin Red S-stained images were analysed by a blinded examiner, using Image J (Fiji) software (the National Institutes of Health (NIH),USA) to quantify osteogenic differentiation and mineralization by measuring the percentage of Alizarin Red S positive area in normal and OA groups in at least 3 high quality fields per donor per condition. In 3/12 donors, this number was not reached, and therefore, these were excluded from the analysis.

### Gene expression analysis by RT-qPCR of native SM tissue and P2 cSM-MSCs

Snap-frozen SM tissue samples wrapped in aluminium foil were reduced to powder using a hammer. After lysis of the homogenate with QIAzol Lysis Reagent (79306, Qiagen, Venlo, The Netherlands), total RNA was extracted using the RNeasy Mini Kit (74104, Qiagen) according to the manufacturer’s instructions, including an on-column DNase step. Total RNA of P2 cSM-MSCs and differentiated cSM-MSCs was isolated using the RNeasy Micro kit (74004, Qiagen), after lysis with RLT buffer containing 1 mg/ml 2-mercaptoethanol (M3701, Sigma-Aldrich, Saint Louis, USA). RNA quality and quantity were measured with a Bioanalyzer (Agilent RNA 6000 Nano Kit, Agilent Technologies, Amstelveen, The Netherlands). Subsequently, cDNA was produced using the iScript™ cDNA Synthesis Kit (Bio-Rad, Lunteren, The Netherlands) with a similar RNA input for all samples (SM tissue: 200 ng, P2 cSM-MSCs: 350 ng, osteogenic differentiated cSM-MSCs: 300 ng, adipogenic differentiated cSM-MSCs: 100 ng), following manufacturer's instructions.

Quantitative RT-PCR was performed using IQ SYBR Green SuperMix and a CFX384 Touch™ Real-Time PCR Detection System (Bio-Rad) according to the manufacturer’s protocols. Pathways related to (synovial) inflammation, the chondrogenic, adipogenic, and osteogenic lineage, and SM and MPC markers were investigated using canine-specific primers (Table 1). Gene expression of MPC markers was included because canine-specific antibodies were not available for all common MPC CD markers. Relative expression was estimated using the efficiency-corrected delta–delta Ct (ΔΔCt) method, employing 7 reference genes (Table 1). If the mean Cq value of reference genes was above 35, the sample was excluded.

**Table 1:** RT-qPCR primers

Category	Gene	Primer sequence	Annealing temperature (°C)	Accession number
Synovial membrane	*PRG4*	F: CCCATATACTTGCTGCTCCT	60	XM_038671150
		R: GCATCTCTAGAATACCCTTCCC		XM_038564806
	*CD55*	F: GCTTCACCCTGATTGGAGAG	60	XM_022420852.1
		R: CTGTAGAAGTCTGAGAACCTCTG		
	*HAS2*	F: TTGACCCTGCCTCATCTG	59	XM_539153.4
		R: AGCCATCCAGTATCTCACA		
Alarmins	*S100A8*	F: GCCATAAACTCCCTCATTGAG	63	NM_001146144.1
		R: ACTCTTGGAACCAGGTGTC		
	*S100A9*	F: GAGACCATCATCAACATCTTCC	58	XM_005622827.1
		R: TGATCTTGTTTATGGCGTTGTC		
MMPs	*MMP-3*	F: CCCAAGTGGAGGAAAACTCA	60	NM_001002967
		R: CACCTCCTTCCAGACATTCAG		
	*MMP-9*	F: CGCATGACATCTTCCAGTACCA	63	NM_001003219
		R: CCGAGAATTCACACGCCAGTA		
Cytokines/chemokines	*IL-1B*	F: TGCTGCCAAGACCTGAACCAC	68	NM_001037971
		R: TCCAAAGCTACAATGACTGACACG		
	*IL-6*	F: GAGCCCACCAGGAACGAAAGAGA	65	NM_001003301
		R: CCGGGGTAGGGAAAGCAGTAGC		
	*IL-8*	F: CTGTTGCTCTCTTGGCAGC	63	XM_850481
		R: GGGATGGAAAGGTGTGGAG		
	*IL-18*	F: GAGGATATGCCCGATTCTGA	56	XM_038664075.1
		R: TCCGGAGGACTCATTTCTG		XM_038664074.1
	*CCL2*	F: AGCCAGATGCAATTATTTCTCC	60	NM_001003297.1
		R: GACGGTCTTGAAGATCACAG		
	*COX2*	F: TTCCAGACGAGCAGGCTAAT	60	NM_001003354
		R: GCAGCTCTGGGTCAAACTTC		
Reference genes	*HPRT*	F: AGCTTGCTGGTGAAAAGGAC	58	NM_001003357
		R: TTATAGTCAAGGGCATATCC		
	*RPL13*	F: GCCGGAAGGTTGTAGTCGT	61	XM_003432726
		R: GGAGGAAGGCCAGGTAATTC		
	*RPS5*	F: TCACTGGTGAGAACCCCCT	62	XM_533568
		R: CCTGATTCACACGGCGTAG		
	*RPS19*	F: CCTTCCTCAAAAAGTCTGGG	62	XM_005616513
		R: GTTCTCATCGTAGGGAGCAAG		
	*SDHA*	F: GCCTTGGATCTCTTGATGGA	61	DQ402985
		R: TTCTTGGCTCTTATGCGATG		
	*TBP*	F: CTATTTCTTGGTGTGCATGAGG	57	XM_849432
		R: CCTCGGCATTCAGTCTTTTC		
	*YWHAZ*	F: CGAAGTTGCTGCTGGTGA	58	XM_843951
		R: TTGCATTTCCTTTTTGCTGA		
CD markers	*CD29*	F: GATGCCTACAACTCCCTTTCCTCA	58	XM_535143
		R: CATTTTCCCCTGTTCCATTCACC		
	*CD34*	F: TCAGGGCCCCCGACATCTC	65	NM_001003341.1
		R: TCTCTGCTCACCCCTCTGGAAAAA		
	*CD44*	F: CTTCTGCAGATCCGAACACA	60	XM_038423375
		R: GAGTAGAAGCCGTTGGATGG		
	*CD73*	F: CTCCAACACATTCCTTTACAC	61	XM_038684083.1
		R: ACTCAACCTTCAAATAGCCT		XM_038675165.1
	*CD90*	F: CAGCATGACCCGGGAGAAAAAG	63	XM_844606.2
		R: TGGTGGTGAAGCCGGATAAGTAGA		
	*CD105*	F: CATCCTTCACCACCAAGAG	60	XM_038678496.1
		R: CAGATTGCAGAAGGACGG		XM_005625330.4
	*CD146*	F: GGGAATGCTGAAGGAAGG	63	XM_038664662.1
		R: CTTGGTGCTGAGGTTCTG		
	*CD166*	F: AAGCGTCATAAACCAAACAG	61	NM_001313804.2
		R: TATAGCAGAGACATTCAAGGAG		
	*VCAM-1*	F: CTACAAGTCTACATCTCACCCA	58	NM_001003298
		R: TTCCAGAATCTTCCAGCCTC		
Chondrogenic markers	*ACAN*	F: GGACACTCCTTGCAATTTGAG	61	NM_001113455
		R: GTCATTCCACTCTCCCTTCTC		
	*SOX9*	F: CGCTCGCAGTACGACTACAC	62	NM_001002978
		R: GGGGTTCATGTAGGTGAAGG		
	*COL2A1*	F: GCAGCAAGAGCAAGGAC	65	NM_001006951
		R: TTCTGAGAGCCCTCGGT		
	*COL1A1*	F: GTGTGTACAGAACGGCCTCA	61	NM_001003090
		R: TCGCAAATCACGTCATCG		
Osteogenic markers	*SPARC*	F: TCTGTATGAAAGGGATGAGGAC	64	XM_014113053.2
		R: GCTTCTCGTTCTCGTGGA		XM_005619272.4
	*RUNX2*	F: AACGATCTGAGATTTGTGGGC	64	XM_845779
		R: TGTGATAGGTGGCTACTTGGG		
	*BGLAP*	F: CTGATGGTCCTTGCCCT	62	XM_014115322.1
		R: CTTGGACACGAAGGTTGC		
	*PTHR1*	F: GACCACATCCTTTGCTGG	51	NM_001003155
		R: CAAACACCTCCCGTTCAC		
	*ALP*	F: GGCTTCAGAATCTCAACAC	55	XM_005617214.1
		R: AACTTGTCCATCTCCAGC		
Adipogenic markers	*ADIPOQ*	F: AGAGAAAGGAGATGCAGGT	62	NM_001006644.1
		R: CGAACGGTGTACATAGGC		
	*PPARG*	F: ACTGGAATTAGATGACAGCGAC	61	XM_038426360.1
		R: CTTCACATTCAGCAAACCTGG		

F, Forward; R, Reverse

### Flow cytometry of cSM-MPCs and P2 cSM-MSCs

Surface marker expression of normal and OA cSM-MPCs was conducted directly after digestion (*n* = 10 per conditions, 0.5–1 × 10^6^ cells/reaction) and P2 cSM-MSCs (*n* = 8 per condition, 1–2 × 10^5^ cells/reaction). Zombie Violet™ Fixable Viability Kit (423114, BioLegend, San Diego, USA; concentration proprietary) was diluted 1:500 in flow cytometry staining buffer (00-4222-26, Invitrogen) and used at 1 μl/L × 10^4^ cells. Cells were incubated in Zombie Violet™ for 15–30 min at room temperature (RT), protected from light. Subsequently, cells were washed with 400 µL flow cytometry staining buffer (00-4222-26, Invitrogen) and resuspended in 50 µL buffer. At this point, cells were incubated with a combination of antibodies against surface markers CD90, CD44, CD73, CD271, CD34, and CD45 (Table 2) for 15 min at 4 °C in the dark. One reaction per donor was left unstained as a negative control. Following antibody incubation, cells were washed with staining buffer and transferred to 5 mL Falcon^®^ round-bottom polypropylene tubes (352063, Corning Life Sciences) for data acquisition. Data collection was performed with CytExpert Software (Version 2.2.0.97, Beckman Coulter, Brea, USA) on a CytoFLEX S Flow Cytometer (Beckman Coulter) and analysed using CytExpert Software version 2.2.0.97. Firstly, small debris was excluded in forward scatter (FSC)/side scatter (SSC) plots. Then, unstained reactions were used to distinguish between background and specific fluorescence signal of each antibody. Based on these gates, the relative numbers of positively or negatively stained events were determined. Dead and hematopoietic cells were excluded by negatively selecting for Zombie Violet™/CD45-stained events, and percentages of CD90, CD73, CD44, CD34, and CD271 positively stained events were evaluated within the CD45- fraction.

**Table 2:** Antibodies used in flow cytometry

Target	Catalogue No	Manufacturer	Host	Reactivity	Clone	Fluorochrome
CD90	12–5900-42	eBioscience	Rat	Dog	YKIX337.217	PE
CD73	bs-4834R	Bioss antibodies	Rabbit	Human, Mouse, Rat, Dog, Chicken	Polyclonal	FITC
CD271	12–9400-42	Invitrogen	Mouse	Dog, Human, Mouse	ME20.4	PE
CD44	11–5440-42	Invitrogen	Rat	Dog	YKIX337.8	FITC
CD45	48–5450-42	Invitrogen	Rat	Dog	YKIX716.13	eFluor 450
CD34	FAB3346S	R&D	Mouse	Dog	IH6	Alexa Fluor 750

No., Number; PE, R-Phycoerythrin; FITC, Fluorescein isothiocyanate

### Immunohistochemical evaluation

To assess the location of cMPCs in the native SM immunohistochemical staining of the MPC markers, CD90 (ab92574, Abcam), CD73 (LS‑B8284, IHCPlus), CD44 (MA1-10225, ThermoFisher Scientific), CD271 (14-9400-82, eBioscience), and CD34 (bs-0646R, Bioss Antibodies) were performed (extensive protocols provided in Additional file 1: Table S2). For the quantification, images were captured using an Olympus BX51 microscope (Olympus, 100 × magnification). Three to six random regions of interest (ROIs) were captured based on availability and analysed by a veterinary pathologist (SAK). The captured images were imported in the Image ProPlus 6.0 software (Media Cybernetics) to quantify the percentage of DAB-stained area.

### Statistical analyses

Statistical analysis was performed using R Statistics (R version 3.6.3 [24], RStudio version 1.2.5033 [25]). Normality was tested via QQ plots, histograms, and Shapiro–Wilk tests. If the data were normally distributed, linear mixed models were employed. If the data was not normally distributed, a Kruskal–Wallis test and Dunn’s multiple comparison test were used. P values of the RT-qPCR analysis were subjected to corrections for multiple testing (Benjamini–Hochberg false discovery rate). Effect sizes (ES) with 95% confident intervals (CI) were calculated using Hedge’s g (HG) for normally distributed data and Cliff’s delta (CD) for non-normally distributed data. Outcomes were considered relevant if *p* < 0.05 or *p* < 0.1 with a large ES. Furthermore, a difference was considered biologically relevant if the expression was undetectable in one of the groups.

## Results

### The synovial membrane of OA knee joints had a higher synovitis grade and increased expression of inflammation markers

**Fig. 1:**
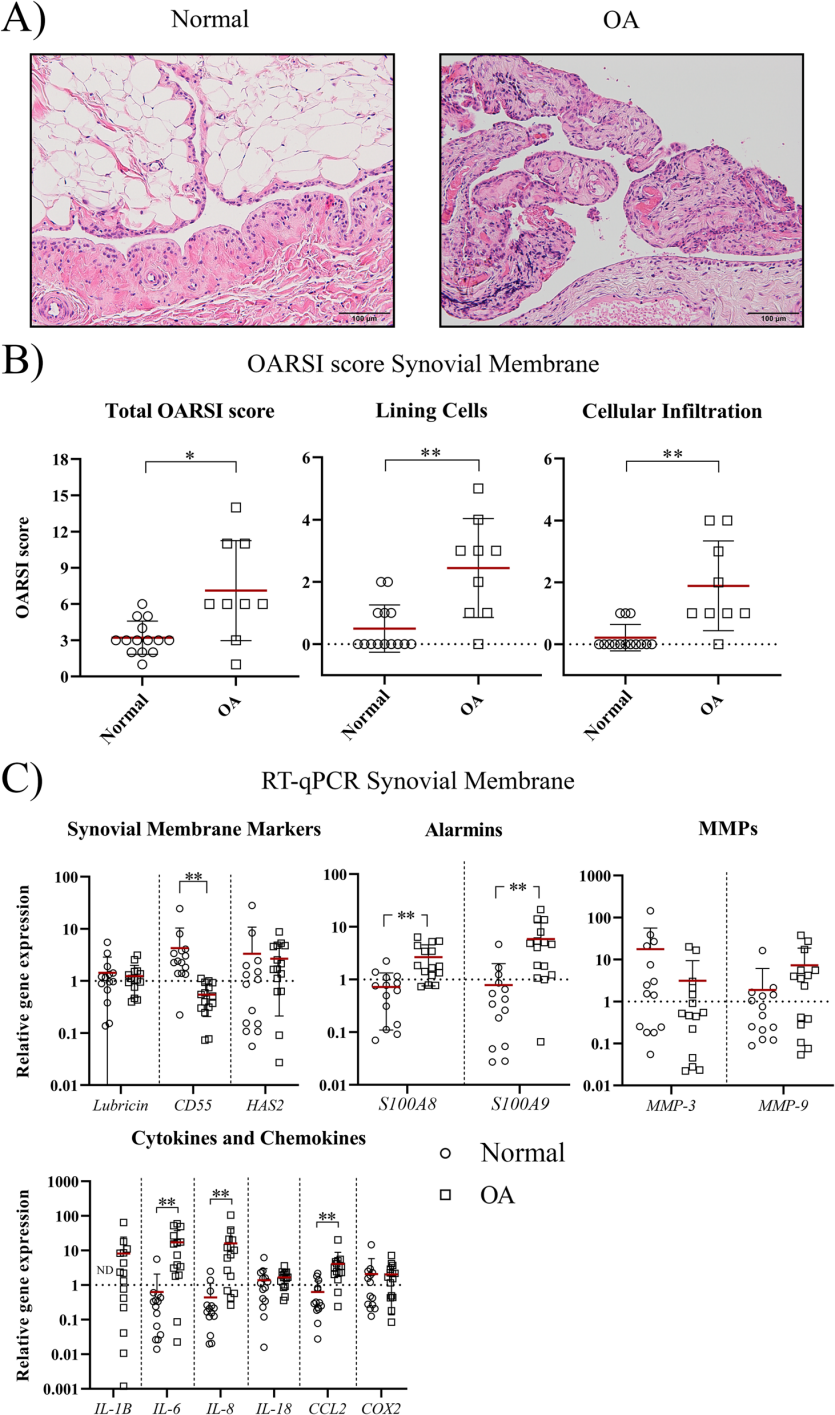
Assessment of inflammation in the synovial membrane. **A** Representative images of normal and osteoarthritic (OA) synovial membrane stained with hematoxylin/eosin. The scale bar is set at 100 µM. **B** OARSI scoring of normal (circle) and OA (square) synovial membrane. The total OARSI score consists of the sum of the individual categories; lining cell characteristics, lining characteristics (not shown), and the evaluation of cellular infiltration. **C** RT-qPCR analysis of markers of the synovial membrane and synovitis. Gene expression is shown on a log scale as the relative gene expression compared to the mean of all samples within a gene. IL-1β was not detected (ND) in the normal samples. Each dot represents an individual donor. *p < 0.05; **p < 0.01

The presence of synovitis in the SM derived from OA knee joints was confirmed by histology. Compared to normal SM, the total OARSI score in the OA SM was increased (*p* = 0.018, ES (HG): 1.4), defined by increased cell numbers in the synovial lining (*p* = 0.0016, ES (CD): -0.8) and increased infiltration of inflammatory cells (*p* = 0.0046, ES (CD): -0.7) (Fig. 1A, B). At the transcriptional level, the synoviocyte marker *CD55* was less expressed in the OA as compared to normal SM (*p* < 0.0001, ES (HG): 1.8). Furthermore, the gene expression levels of pro-inflammatory cytokines *IL-1β, IL-8*, and *IL-6,* the chemokine *CCL2,* and the synovitis markers *S100A8* and *S100A9* were higher in the OA SM (*p* < 0.0001, ES (HG) > 1.5) (Fig. 1C). The synovial membrane markers *Lubricin* and *HAS2*, and the inflammation markers *MMP3,* and *-9, IL-18*, and *COX2* did not differ between groups.

### Cell culture

#### OA cSM-MPCs possess a lower CFU capacity

**Fig. 2:**
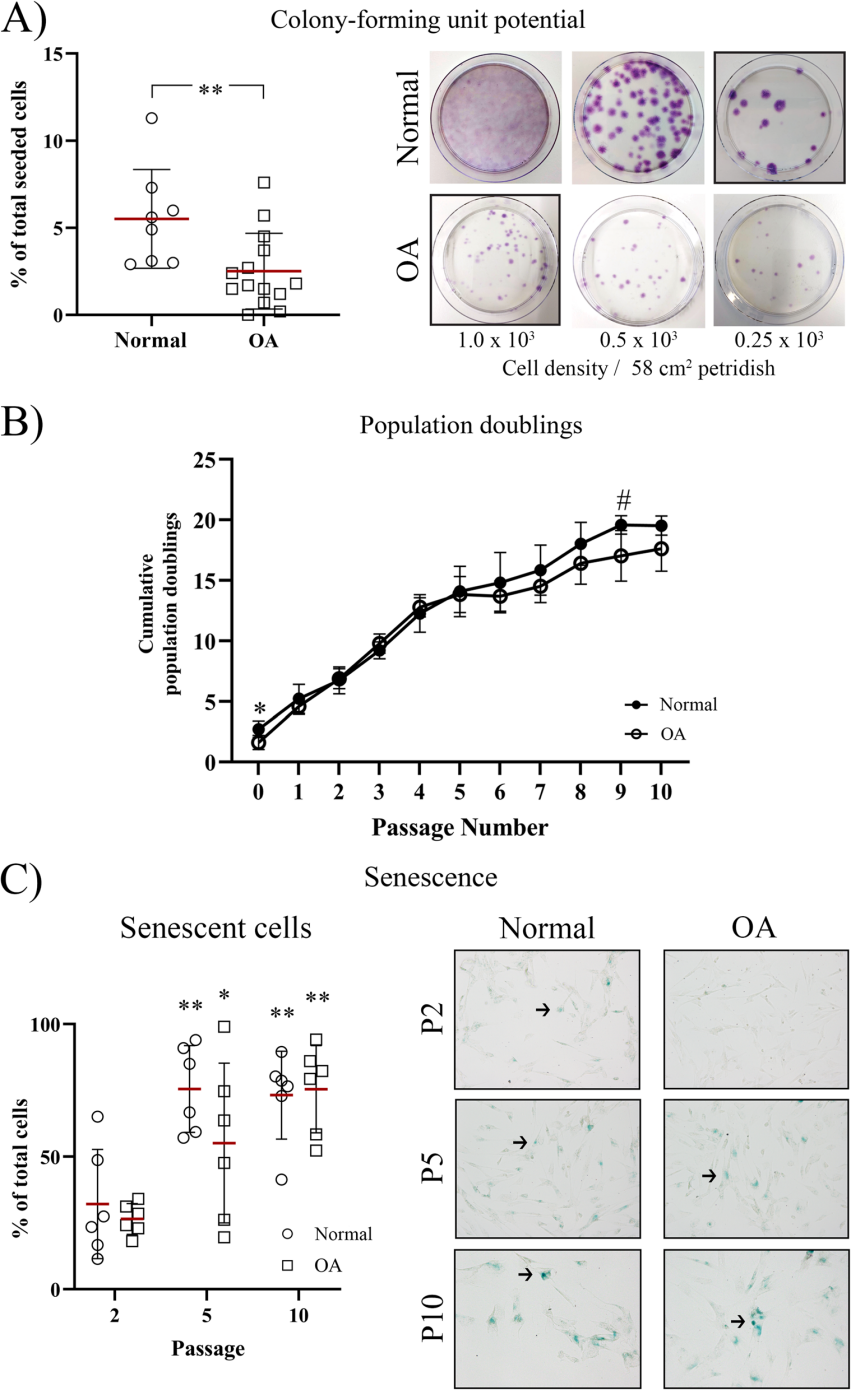
Cell culture characteristics. **A** Colony-forming unit (CFU) potential. The optimal seeding cell density was determined for normal (circle) and osteoarthritic (OA, square) donors by testing three densities. For OA donors, a cell density of 1.0*10^3^ was chosen, and for the normal donors a cell density of 0.25*10^3^. The amount of CFUs was displayed as the percentage of CFU of the total seeded cells. **B** Population doublings are displayed as the cumulative population doublings per passage for normal (black dots) and OA (clear dots). # *p* < 0.15 with a large effect size and *p < 0.05 in the normal compared to the OA cSM-MSCs **C** Senescence of the normal and OA cSM-MSCs was investigated using a β-galactosidase assay in passage (P) 2, 5, and 10. The number of senescent cells (stained blue in the microscopic images (black arrows)) was depicted as the percentage of senescent cells of the total cells. Each dot represents an individual donor. *p < 0.05; **p < 0.01 compared to the percentage of senescent cells at P2

The optimal density for the CFU assay was 0.25 × 10^3^ cells/petri dish for the normal cSM-MPCs and 1 × 10^3^ cells/petri dish for the OA cSM-MPCs (Fig. 2A). A lower percentage of CFU forming cells was found for OA compared to normal cSM-MPCs (*p* = 0.01, ES (CD): 0.7) (Fig. 2A).

Upon culture and expansion, normal and OA cSM-MSCs showed a fibroblast-like morphology. After P5, population doublings declined in normal and OA cSM-MSCs (Fig. 2B, Additional file 1: Fig. S2). The cumulative population doublings were lower at P0 (*p* = 0.013, ES (HG): 1.6) and P9 (*p* = 0.12, ES (HG): 1.5) for OA compared to normal cSM-MSCs (Fig. 2B).

Normal and OA cSM-MSCs had a similar percentage of senescent cells at all passages. The percentage of senescent cells was increased at P5 and P10 as compared to P2 for normal (P5 and P10: *p* = 0.0012, ES (HG) > 2.0) and OA cSM-MPCs (P5: *p* = 0.02, ES (HG): 1.2; P10: *p* = 0.0003, ES (HG): 3.7) (Fig. 2C).

#### Marker expression upon expansion at P2 is comparable between OA and normal cSM-MSCs

**Fig. 3:**
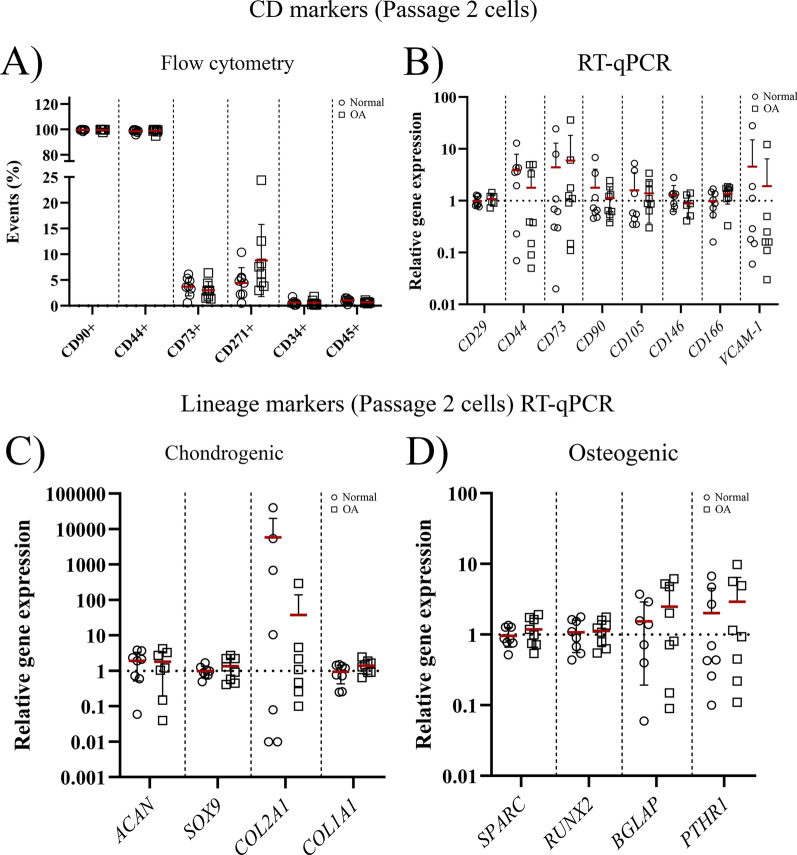
Marker expression in passage 2 (P2) cultured cSM-MSCs. **A** Evaluation of CD markers by flow cytometry in P2 cultured normal (circle) and OA (square) cSM-MSCs. Expression is shown as the percentage (%) of positive cells (events) of all live cells for CD90, CD44, CD73, CD271, CD34, and CD45. RT-qPCR analysis of **B** CD marker expression, **C** chondrogenic lineage and **D** osteogenic lineage markers in P2 cultured cSM-MSCs. Gene expression of the markers is shown on a log scale as the relative gene expression compared to the mean of all samples within a gene. Each dot represents an individual donor

Upon expansion in vitro, the CD marker expression profile of MSCs shifts towards the MSC marker expression reported by the ISCT [14, 26]. CD markers and the expression of chondrogenic and osteogenic genes were measured in P2 expanded cells. On flow cytometry, both normal and OA P2 cSM-MSCs showed a high expression (~ 99%) of CD90 and CD44, variable expression of CD73 and CD271 and undetectable expression (< 1%) of CD34 and CD45 (Fig. 3A). At the transcriptional level, gene expression of MPC/MSC markers was detectable, but without differences (Fig. 3B), and expression of chondrogenic and osteogenic markers between the cultured, undifferentiated normal and OA cSM-MSCs did not differ (Fig. 3C, D).

#### Tri-lineage differentiation capacity

**Fig. 4:**
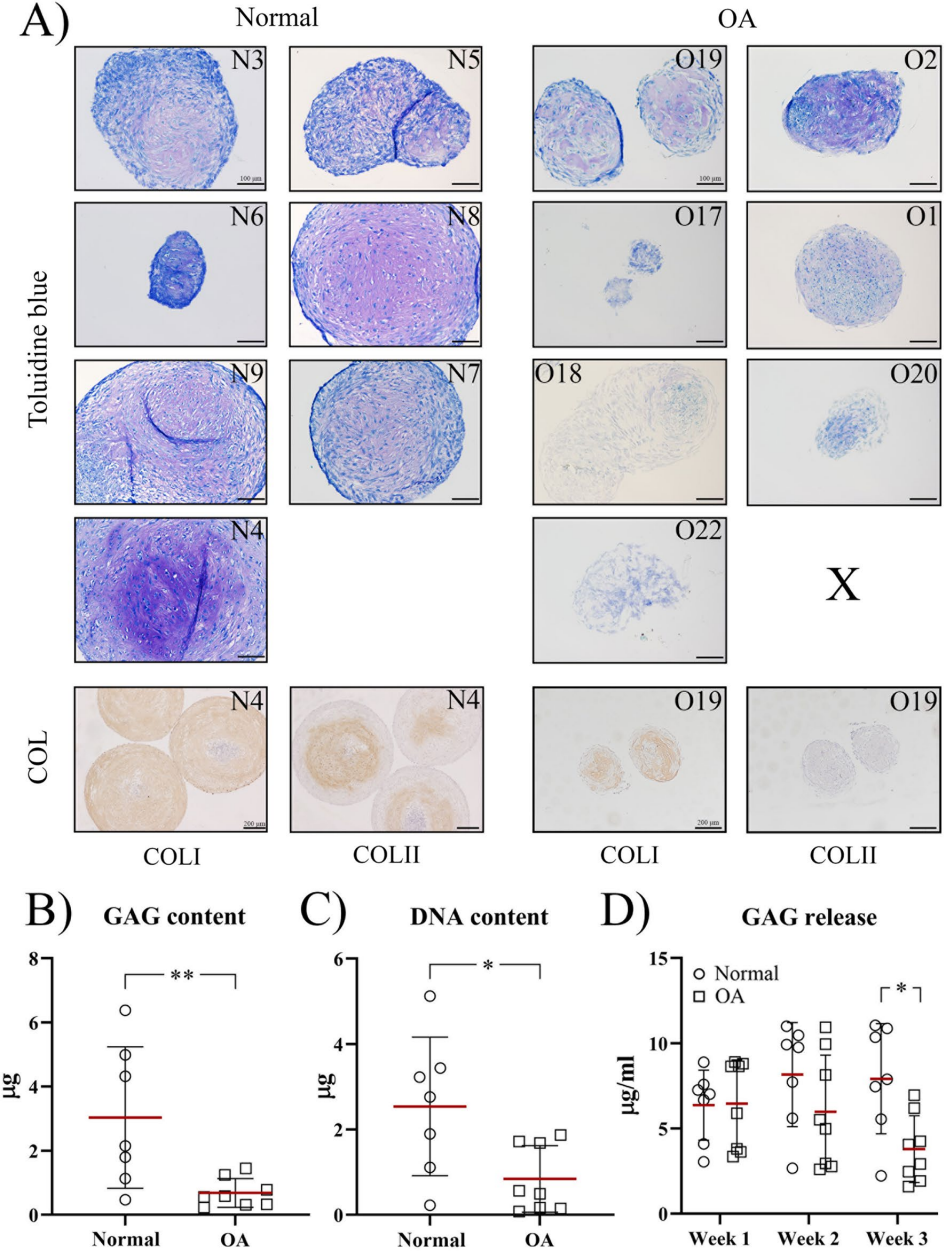
Chondrogenic differentiation of normal and osteoarthritic (OA) synovial membrane-derived cMSCs. **A** Evaluation of glycosaminoglycan (GAG) deposition and collagen content. 5 µm sections of pellets cultured for 21 weeks in chondrogenic differentiation medium containing 10 ng/ml TGF-β1 and 100 ng/ml BMP-2 were stained with toluidine blue (scale bar = 100 µM). A representative image of every donor is shown. One donor (X) was lost during processing. Additionally, immunohistochemical analysis of collagen (COL) content was performed for COL type I and II using DAB (3, 3'-diaminobenzidine, orange/brown staining) (scale bar = 200 µm). The donor numbers of clinically normal (N) and OA (O) donors correspond to the donor numbers and information in Additional file 1: Table S1. Biochemical evaluation of the GAG (**B**) and DNA (**C**) content in µg. Each dot represents an individual donor. *p < 0.05; **p < 0.01

The tri-lineage differentiation potential was assessed to investigate whether the cSM-MSCs lose their multipotent differentiation potential due to the OA environment and determine their chondrogenic potential, an asset for cartilage regenerative strategies. Under chondrogenic culture conditions, at day 21 the DNA content of the OA cSM-MSC pellets was lower (*p* = 0.043, ES (HG): 1.3) compared to normal cSM-MSC pellets. 4/7 cSM-MSC donors from normal donors differentiated towards the chondrogenic lineage based on the toluidine staining for GAG deposition and the presence of chondrocyte-like cells (Fig. 4A). 1/7 normal cSM-MSC donors showed COL2 immunopositivity of the extracellular matrix. cSM-MSCs of two of the OA donors showed a very mild positive toluidine blue but none of them showed COL2 immunostaining. These observations were line with the lower GAG content and GAG release in the third week of culture in the OA compared to normal cSM-MSCs (*p* = 0.0054, ES (HG): 1.4) (Fig. 4B). cSM-MSC pellets from all normal and OA donors showed variable staining for COL1.

**Fig. 5:**
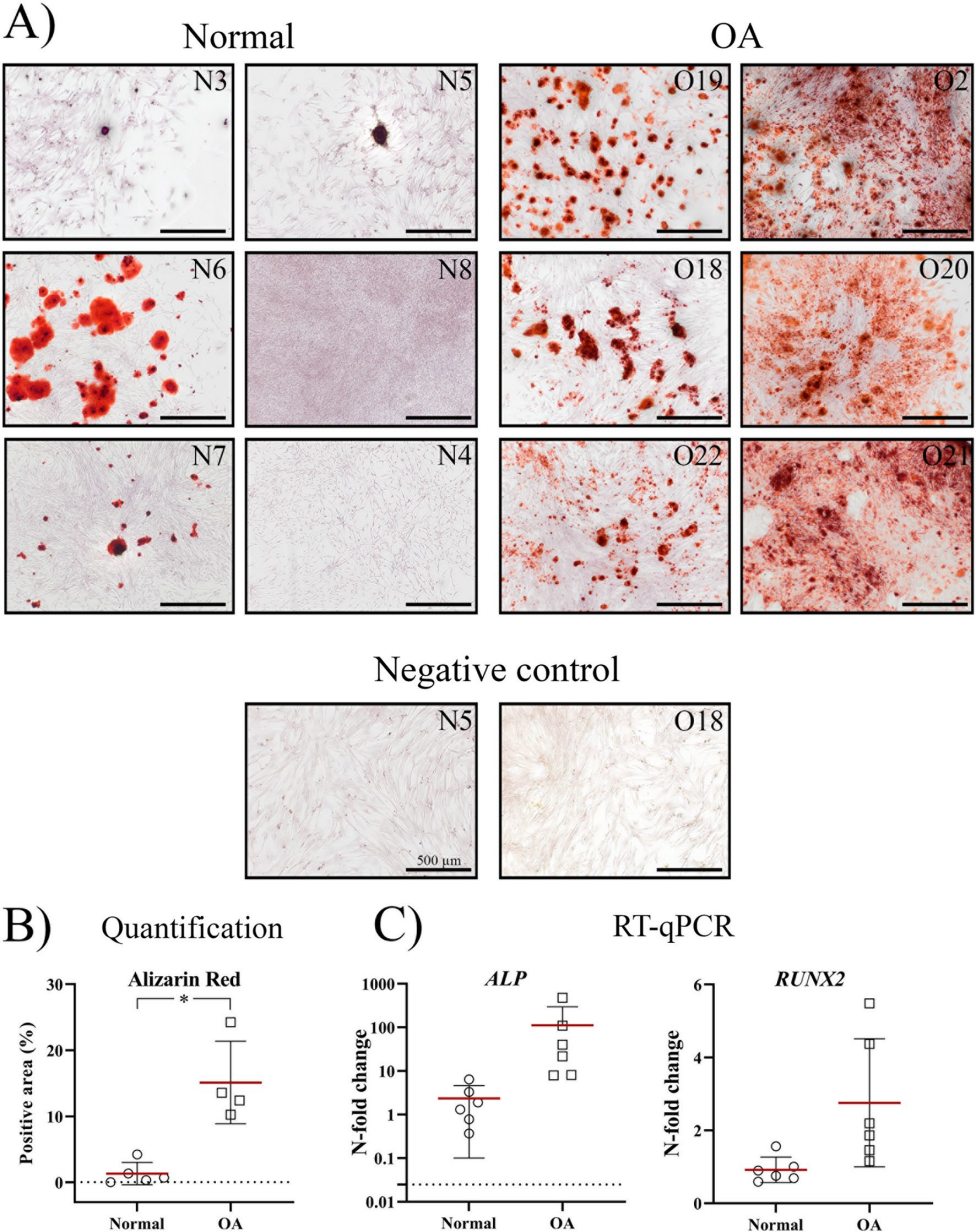
Evaluation of osteogenic differentiation by Alizarin Red staining. **A** Calcium (stained in red) and the presence of noduli are representative of successful osteogenic differentiation. Representative images of every donor were obtained using the brightfield setting of an Olympus IX51 inverted microscope. The negative controls received expansion medium for 21 days. The scale bar is set at 500 µm. The donor numbers of clinically normal (N) and OA (O) donors correspond to the donor numbers and information in Additional file 1: Table S1. **B** The percentage (%) of Alizarin Red positive area was measured using Image J (Fiji) software in at least 3 image per donor per condition. **C** Gene expression of the osteogenic markers alkaline phosphatase (*ALP*) and *RUNX2* is shown as the N-fold change of the cells treated with osteogenic differentiation medium compared to their own negative control. Each dot represents an individual donor

Alizarin Red positive noduli, indicative for osteogenic differentiation, were found in cSM-MSCs from 4/6 normal donors, although in two donors only a few noduli were observed (Fig. 5). In contrast, in cSM-MSCs from all OA donors mineralized matrix was deposited with intense Alizarin Red staining, indicating osteogenic capacity. The negative controls were devoid of Alizarin Red stain. Quantification of the staining showed a significant higher percentage of Alizarin Red positive stained area in the OA cSM-MSCs compared to the normal cSM-MSCs (*p* = 0.019, ES (HG): 3.7). Gene expression analysis of *ALP* and *RUNX2* showed no differences between the osteogenically differentiated normal and OA cSM-MSCs.

**Fig. 6:**
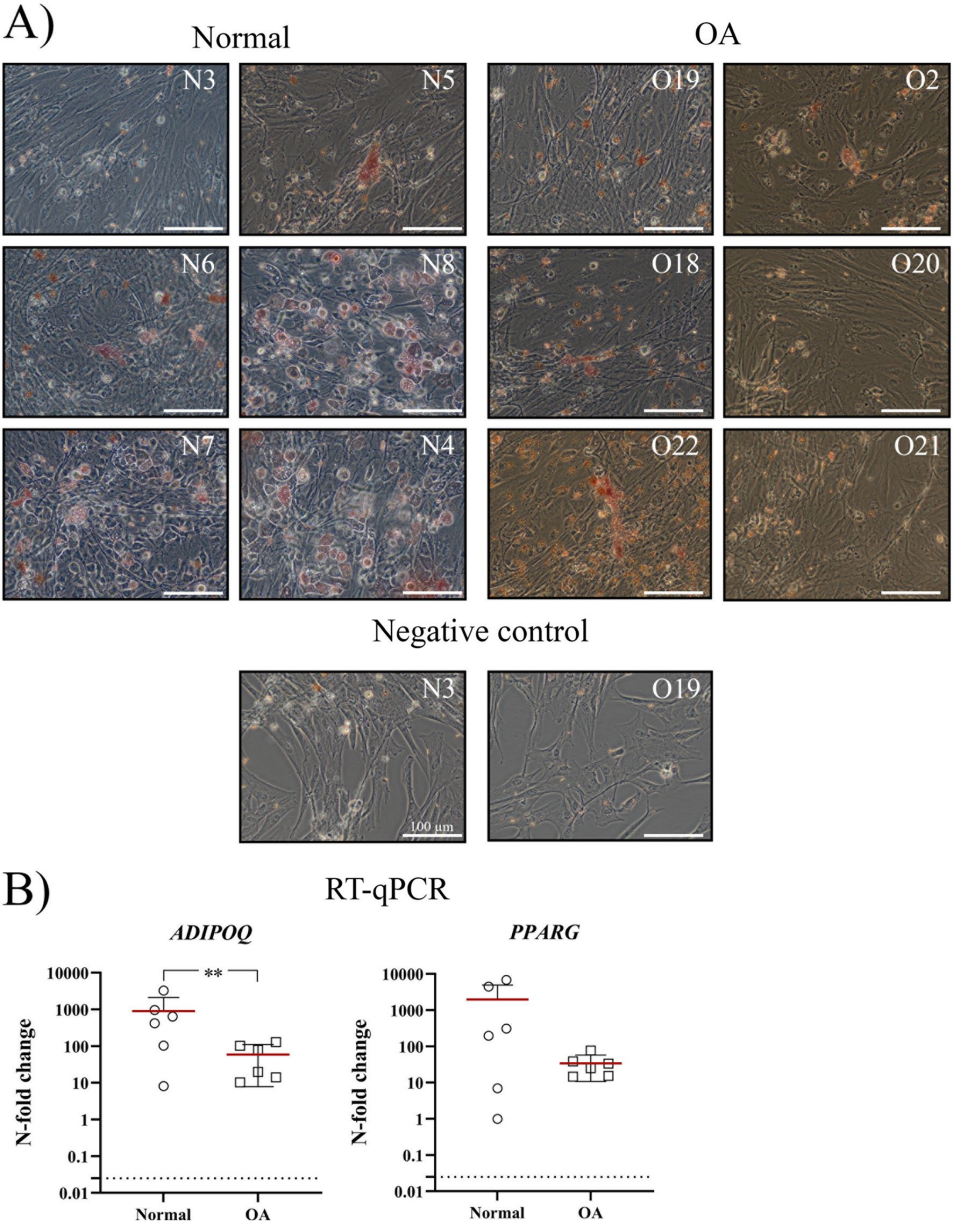
Evaluation of adipogenic differentiation by Oil Red O staining. Lipid droplets (stained in red) and the more rounded cell morphology are representative of successful adipogenic differentiation. Representative images of every donor were obtained using the phase contrast setting of an Olympus IX51 inverted microscope. The negative controls received expansion medium for 21 days. The scale bar is set at 100 µm. The donor numbers of clinically normal (N) and OA (O) donors correspond to the donor numbers and information in Additional file 1: Table S1. **B** Gene expression of the osteogenic markers adiponectin (*ADIPOQ*) and peroxisome proliferator-activated receptor gamma (*PPARG)* is shown as the N-fold change of the cells treated with osteogenic differentiation medium compared to their own negative control. Each dot represents an individual donor. *p < 0.05

cSM-MSCs from normal donors showed a more successful adipogenic differentiation as they had a higher number of cells with a rounded morphology and Oil Red O-stained lipid droplets compared to the cSM-MSCs from OA donors (Fig. 6). Expression of *ADIPOQ* was significantly higher in the adipogenic differentiated normal cSM-MSCs compared to the OA cSM-MSCs (*p* = 0.0067, ES (HG): 1.7), while *PPARG* was not significantly different between the two groups.

### CD marker expression in the synovial membrane

**Fig. 7:**
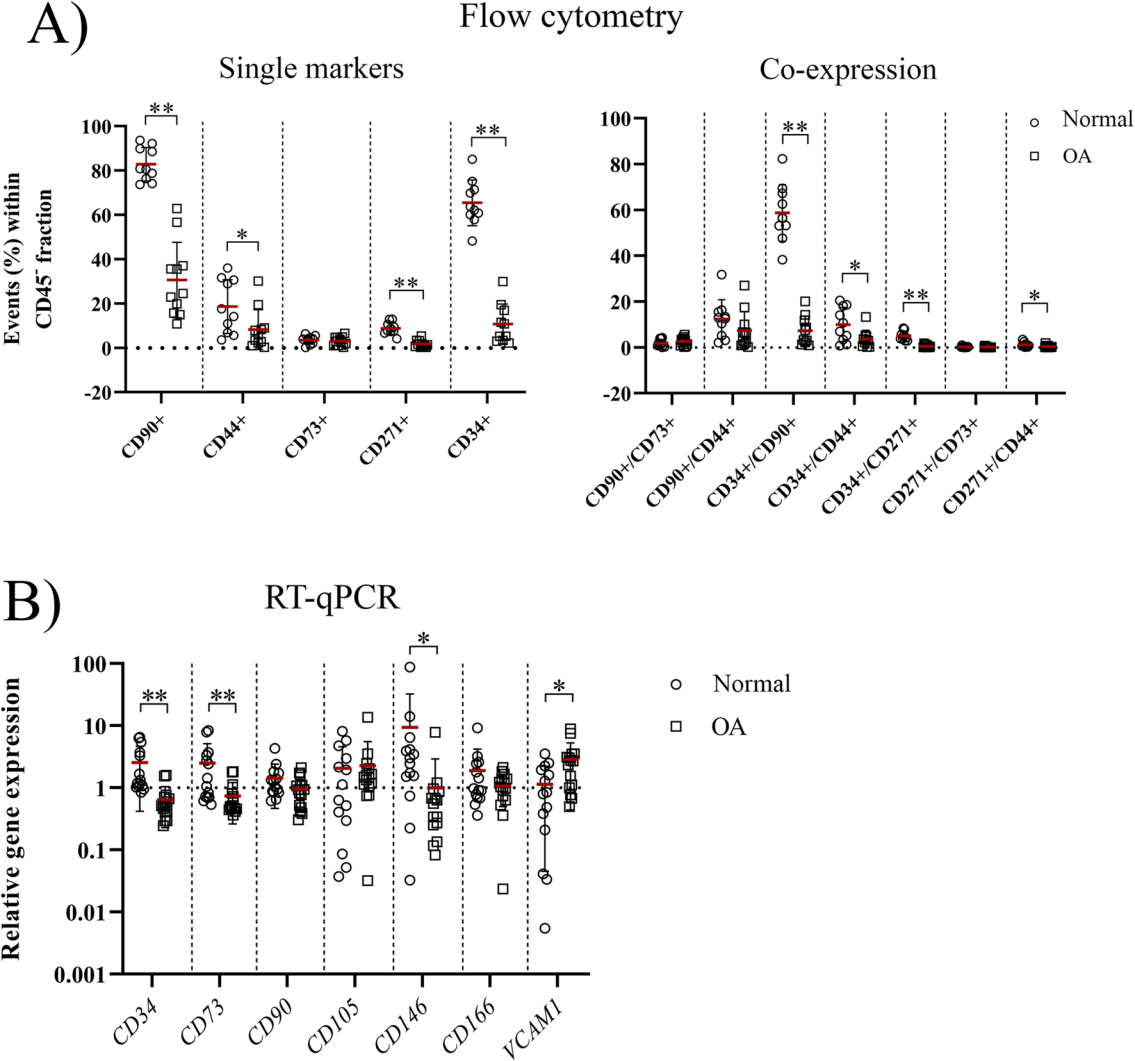
Quantitative evaluation of CD marker expression in the synovial membrane. **A** Evaluation of CD markers by flow cytometry. Expression is shown as the percentage of positive cells (events) within the CD45 negative cell population for each single marker and for the co-expression of markers for normal (circle) and osteoarthritic (OA, square) synovial membrane. **B** RT-qPCR analysis of CD marker expression in the synovial membrane. Gene expression of the CD markers is shown on a log scale as the relative gene expression compared to the mean of all samples within a gene. Each dot represents an individual donor. *p < 0.05; **p < 0.01

**Fig. 8:**
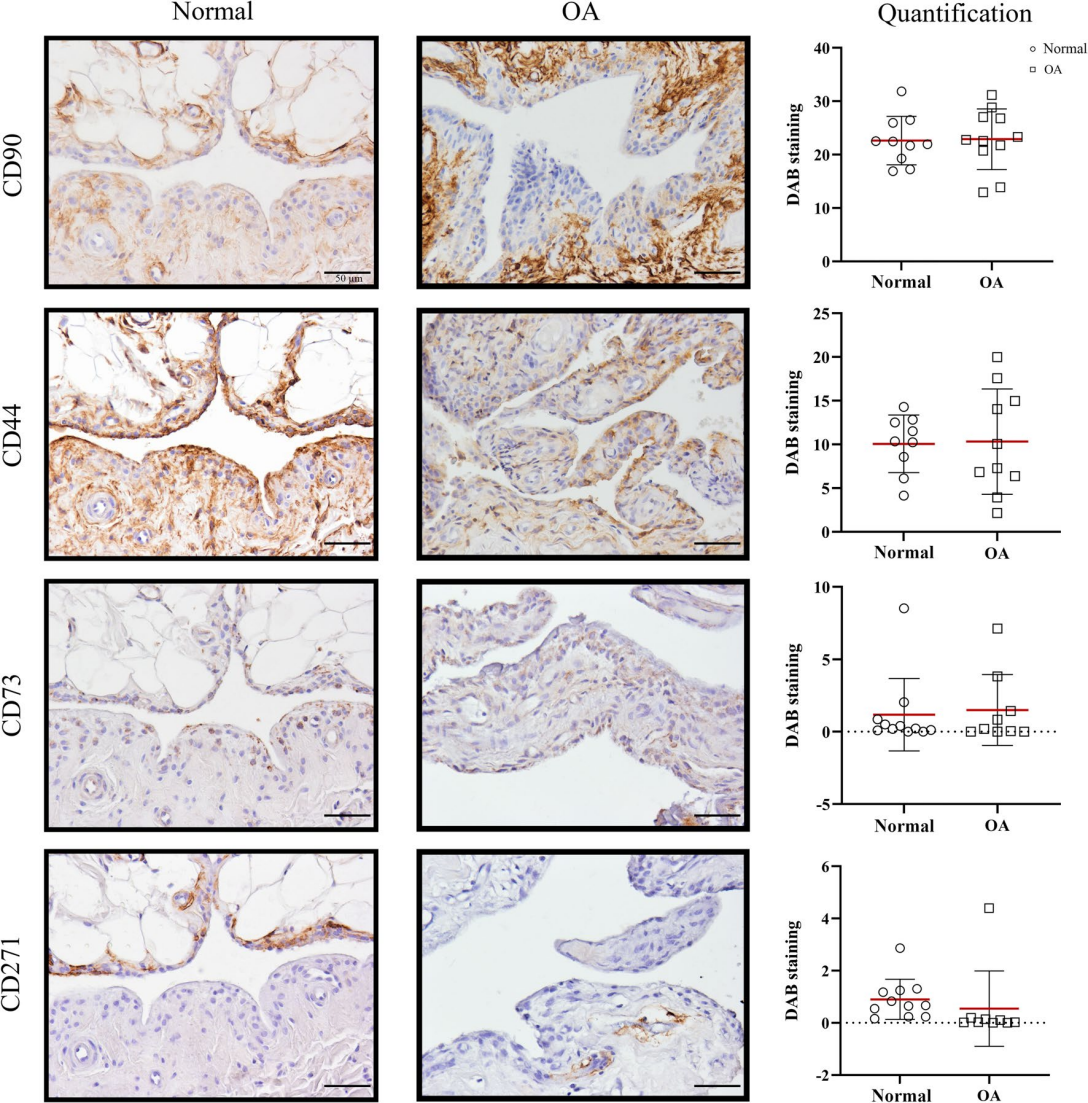
Evaluation of the CD marker location in the synovial membrane. Immunohistochemical analysis using DAB (3, 3'-diaminobenzidine, orange/brown staining) was performed to identify the location of CD90, CD44, CD73, CD271, and CD34 expression in the normal (circle) and osteoarthritic (OA, square) synovial membrane. Representative images, containing both fibrous and adipose type synovial membrane, were chosen. The scale bar is set at 50 µm. Quantification of the DAB staining was performed with Image ProPlus 6.0 software (Media Cybernetics) in 3 to 6 random regions of interest (ROI) per donor, resulting in a mean percentage of DAB positive staining areas for each donor

**Fig. 9:**
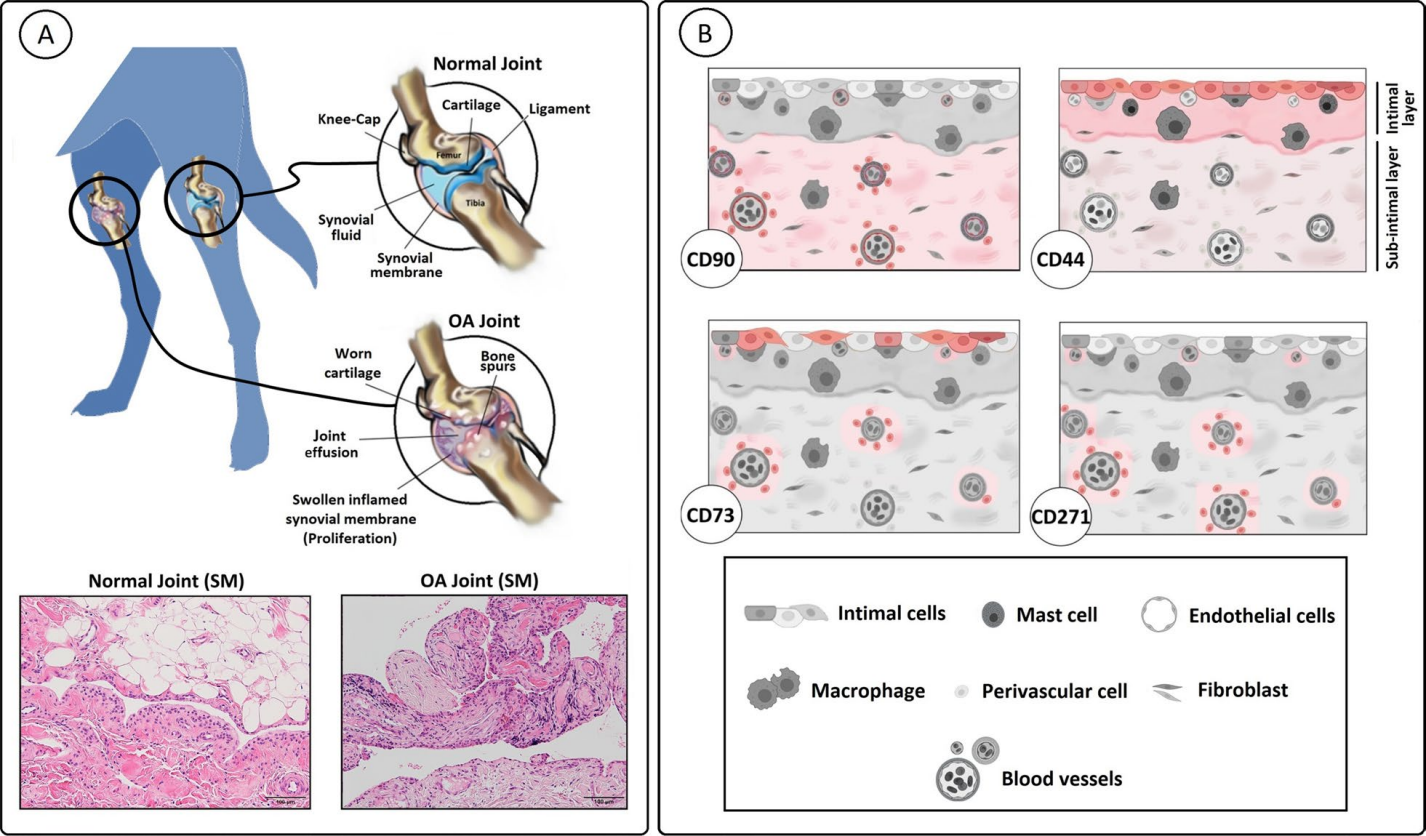
Graphical overview of the CD marker expression in the synovial membrane. **A** Synovial membrane (SM) was collected from normal and OA knee joints **B** Immunohistochemical staining was performed for CD90, CD44, CD73, and CD271. Tissue expression of these markers is visualized in red. Immunopositivity of CD90 was observed mainly in the subintimal and perivascular layer. CD44 was mainly expressed in the intimal lining cells, although variable expression was observed in the subintimal layer and perivascular. CD73 positive cells were mostly observed in the intimal lining and perivascular layer. CD271 was expressed perivascular and in the normal SM also in the intimal layer of the adipose parts of the synovial membrane

As cSM-MSCs from normal and OA knee joints differed in their tri-lineage potential and cell culture is known to influence CD marker expression profiles [14], follow-up work focused on studying the cSM-MPCs in situ in the SM of normal and OA joints. For this purpose, the presence of MPC markers was investigated using RT-qPCR on snap-frozen SM tissue, flow cytometry on freshly isolated cells from SM tissue digest and complemented with immunohistochemical stains of corresponding paraffin-embedded tissue. These complementary results are described below per CD marker (Figs. 7, 8) and in a graphical representation (Fig. 9).

#### CD90

Within the CD45- cell population, the percentage of CD90+ cells decreased in the OA compared to normal SM (*p* < 0.0001, ES (CD): 1.0) (Fig. 7A). In line with this, a trend towards a lower gene expression of *CD90* was found in the OA SM (*p* = 0.08, ES (HG): 0.7) (Fig. 7B). Immunopositivity of CD90 was observed mainly in the subintimal and perivascular layer while the intimal layer was almost completely CD90 negative, with no distinct spatial differences between the normal and the OA SM (Figs. 8, 9).

#### CD44

Flow cytometry demonstrated decreased percentages of CD44+ cells in the OA SM (*p* = 0.026, ES (HG): 0.9) in the CD45- cell population compared to normal (Fig. 7A). On immunohistochemistry, CD44 was mainly expressed in the cell membrane of the intimal lining cells, although some expression was also variably observed in the subintimal layer and perivascular (Figs. 8, 9).

#### CD73

There was no significant difference in the percentage of CD73+ cells in OA compared to normal SM, using flow cytometry (Fig. 7A). However, gene expression of *CD73* was significantly lower in the OA SM (*p* = 0.0046, ES (CD): -0.7) (Fig. 7B). CD73 positive cells were mostly observed in the intimal lining and perivascular layer of the synovial membrane with high variability in staining between donors for both normal and OA SM (Figs. 8, 9).

#### CD271

The most distinct difference between normal and OA SM was observed for CD271: in the OA SM there were less CD271+ cells (*p* < 0.0001, ES (HG): 3.0) compared to normal SM (Fig. 7A). In addition, the double-positive, CD271+/CD44+ fraction of cells was decreased in the OA SM (*p* = 0.02, ES (CD): 1.0) (Fig. 7A). Noticeable diversity in CD271 expression was observed on immunohistochemistry between OA and normal SM (Fig. 8). In the OA SM, CD271 was only limited expressed in perivascular regions in the connective tissue layers adjacent to the SM. In normal SM, CD271 expression was abundant in the adipose parts of the synovial membrane and commonly observed in the intimal layer and the perivascular regions (Fig. 9).

#### CD34

Within the CD45- cell population, the CD34+ fraction was lower in the OA SM than in the normal SM (*p* < 0.0001, ES (CD): 1.0) (Fig. 7A). Interestingly, almost all double-positive populations expressing CD34+ were lower in the OA SM compared to normal SM (CD34+/CD90+: *p* < 0.0001, ES (CD): 1.0; CD34+/CD44+: *p* = 0.048, ES (HG): 1.0; CD34+/CD271+: *p* = 0.0003, ES (CD): 1.0) (Fig. 7A). This was confirmed on RT-qPCR; OA SM tissue expressed lower levels of *CD34* gene expression compared to normal SM (*p* < 0.0001, ES (HG): 1.8) (Fig. 7B).

#### *CD146, VCAM1* and* CD166*

These markers are not available as canine-specific antibody for flow cytometry and were therefore only evaluated at the transcription level. RT-qPCR showed decreased *CD146* (*p* = 0.024, ES (HG): 1.0), and increased *VCAM1* (*p* = 0.024, ES (CD): 0.5) in the OA compared to normal SM (Fig. 7B). No difference was found for the expression of *CD166* in the OA compared to the normal SM.

## Discussion

This study aimed to isolate and characterize canine mesenchymal progenitor/stromal cells from normal and OA canine synovial membrane tissue to study the effect of the OA environment. For this purpose, surface marker expression profiling of the cMPCs in fresh SM digest and of the cultured P2 cells (referred to as cMSCs) and tri-lineage differentiation assays of cSM-MSCs were conducted. This is the first time that the tri-lineage differentiation capacity of cSM-MSCs is directly compared between OA and normal joints. The isolated cMPCs and cMSCs from normal and OA SM possessed many of the characteristics that are described for MSC by the International Society for Cellular Therapy (ISCT)[26]; a fibroblast-like morphology on culture plastic, CFU capacity, positivity for CD90, CD73, and CD44 without expression of CD45 and CD34, and tri-lineage differentiation capacity. However, distinct functional differences were observed, depending on the health state of the tissue they were derived from, being more distinct at the CD marker level in the cSM-MPCs rather than the cultured P2 cSM-MSCs. As the CD marker expression is known to change during culture [14, 27], the absence of distinct differences in the P2 is most probably due to effects of the expansion procedure. cSM-MSCs have been described to outperform other MSC tissue sources like bone marrow and adipose tissue in chondrogenic capacity [19, 20]. In this study, normal cSM-MSCs showed a quite heterogeneous chondrogenic differentiation capacity, with only one donor showing deposition of collagen type II. Variation in chondrogenic capacity between donors is well reported [28], while the use of super-physiological concentrations of growth factors may lead to a more robust chondrogenic differentiation and pellet maturation. Nonetheless, under the same culture conditions, the cSM-MSCs from OA joints displayed lower chondrogenic and adipogenic, but better osteogenic capacity compared to cSM-MSCs from normal joints. This coincides with the findings of a direct comparison between pre-OA and OA human SM-MSCs [11] in which OA SM-MSCs possessed lower CFU capacity, a lower proliferation rate, a higher percentage of senescent cells, and lower chondrogenic differentiation capacity. Moreover, this impaired chondrogenic capacity of SM-MSCs was also reported in sheep, in which the inflammatory joint environment, created by transecting the anterior cruciate ligament, was related to a decreased cartilage pellet size and GAG deposition [29]. Furthermore, the catabolic factors in conditioned medium derived from the OA synovial membrane inhibited the chondrogenic differentiation of human MSCs in vitro [30, 31]. Altogether, this implies that the OA joint environment has a negative impact on the chondrogenic capacity of joint MPCs and MSCs generated upon MPC expansion.

Interestingly, cSM-MSCs derived from the OA joints had a better osteogenic capacity than cMSCs from normal joints. This is in line with the increased osteogenic capacity of cSM-MSCs derived from OA joints with cruciate ligament disease compared to cSM-MSCs from OA joints with medial patella luxation [21]. The former OA joints typically have more severe synovial membrane inflammation while the latter come with only mild synovitis [21]. This contrasts with earlier studies, which reported no difference in osteogenic capacity between normal and OA human bone marrow-[10] and ovine SM-MSCs [32]. However, the effect of inflammatory cytokines on the osteogenic differentiation of MSCs is ambiguous. For example, TNFα has been reported to inhibit as well as promote osteoblastogenesis, depending on the cell type, the animal model, and the timing, duration, and dosage of TNFα administration [33, 34]. While we cannot exclude that this contradiction could also be species-dependent, the available literature implies that the levels of inflammation within the OA environment may differentially prime the heterogeneous MPC population and affect their osteogenic capacity.

The decreased CFU capacity and decreased chondrogenic differentiation capacity of cSM-MSCs from OA joints raise the question whether the number of multipotent progenitor cells is exhausted during OA, and/or if their phenotype is altered priming cells towards the osteogenic lineage. Exhaustion and dysfunction of the progenitor cell population has been described for other musculoskeletal tissues with ageing and degeneration, e.g. the intervertebral disc and in fracture bone healing [35, 36]. Additionally, the OA environment might also accelerate the ageing of the SM-MPCs, resulting in a decreased proliferation capacity and increased senescence [37]. Although the exact relationship between senescence and OA is still largely unknown, it is thought that cellular senescence may play a significant role in the pathology of OA [38]. In turn, ageing and senescence have a negative effect on the regenerative capacities of MSCs [39]. In the present study, OA cSM-MSCs had a lower population doubling in P9 compared to normal cSM-MSCs, but senescence levels did not differ from normal, which is in contrast with earlier studies reporting increased senescence in OA SM-MSCs [11]. In light of the differences in senescence of bone marrow-derived MSCs observed between different dog breeds [40] and the large variation observed within this study in senescence, a larger sample size is needed to determine the cofounding role of OA severity in cellular senescence of the cSM-MSCs.

Differences between studies relating to differences in species, OA severity, joint location, and injury type could influence the effect of OA on MPCs in the synovial membrane. Earlier studies used immunostainings to investigate MSC/MPC markers to quantify the presence and spatial distribution of progenitor cells during OA in the synovial membrane. In contrast to this study, they demonstrated an increase in CD90, CD44, and CD271 positive cells in the OA compared to normal synovium [12, 13, 41], suggesting that MPCs increase in numbers in the OA synovium. There are, however, important differences to consider while interpreting these observations. Firstly, immunostainings of tissue sections are not as quantitative as flow cytometry of an entire synovial membrane tissue sample. Secondly, CD90 was reported to be expressed in both the intimal and subintimal lining [12, 13], in contrast to the present study in which CD90 was mainly expressed in the subintimal lining of the canine synovial membrane. This might be a species-dependent difference; in immunostainings of human OA SM tissues with the CD90 antibody used in the present study we observed less intense staining and a CD90 perivascular localisation (Additional file 1: Fig. S3). However, the high and widespread expression and immunopositivity of CD90 raises the question whether it is a good marker for canine MSCs. Finally, OA severity influences CD marker expression: *Del Rey et al.* (2016) reported that while CD271 positivity was not increased in pre-OA samples, with a moderate to severe synovitis, compared to normal synovial tissue, it was increased in OA samples [41]. In line with this, CD271 immunopositivity in our study was highest in one of the dogs with severe OA. Additionally, the joint location (hip versus knee) and injury type might also have an effect. For example, human SM-MSCs derived from the hip joints of patients with femoroacetabular impingement syndrome showed higher proliferative and chondrogenic capacity compared to SM-MSCs derived from patients with hip OA [42]. Furthermore, *Wijekoon et. al.* (2017) showed differences between OA SM-MSCs from dogs with medial patellar luxation and cranial cruciate ligament disease [21].

The use of MSC/MPC markers to determine the relative numbers of progenitor cells in vivo is hampered by the lack of specific markers. While the use of functional markers, such as iododeoxyuridine (IdU) to mark slow-cycling cells [3], or lineage tracing, using important developmental markers such as *Gdf5* [4], are promising techniques, they are not feasible to investigate progenitor cells in humans and large animal models, such as the dog. Therefore, the combination of the known MSC/MPC markers with new or less known markers is necessary in the canine species. In this study, CD34 was used as a marker for progenitor cells and was shown to decrease together with CD90 and CD44. While this marker is often considered a negative marker for MSCs [26], it is a common misconception that all CD34+ cells are hematopoietic [43], and this is presumed because isolated CD34+ MPCs lose their CD34+ positivity upon in vitro culture [43]. As such, CD34 might be an interesting MPC marker to use, although the tissue source should be considered in this context. To date, CD34 is mostly associated with adipose tissue derived MPCs [43]. In this study, the normal canine synovial membrane contained relatively more adipose tissue compared to the OA synovial membrane. Therefore, this may explain the relatively smaller fraction of CD34+ cells detected in OA samples and the decreased adipogenic capacity of the OA cSM-MSCs in this study. New markers seem to be necessary to move forward in the MPC marker field and unbiased methods such as single cell RNA sequencing of the synovium tissue digest could be used to study the heterogeneous cell population in vivo identity of the SM-MPCs and discover new markers.

### Limitations

There are several limitations to this study, due to the design of this study that prioritized working on naturally occurring OA and the choice to work with patient-derived samples instead of experimentally induced OA. The latter results in a studied population that varies in breed, age, and body size, which contributes to the heterogeneity of the results, as all parameters probably affect cMPC characteristics. As a result, a major limitation of this study is that the OA donor group was significantly older compared to the normal group. Normal tissues were collected from experimental dogs euthanized in unrelated experiments that typically employ young adults. Age is known to influence MSC characteristics [44], and although no difference in senescence was found between the two groups, it would have been preferable to have an older control group in which OA is excluded. However, this is a challenge, as up to 80% of the dogs older than 8 years, has radiographic or clinical signs of OA [45]. Therefore, this limitation is almost inevitable when working with patient-derived material. Nevertheless, the use of patient-derived material also has clear advantages, including the possibility to study cSM-MPCs in a more “natural” OA situation, which could increase the translatability of the findings. Furthermore, by using patient-derived material, available from standard-of-care surgical treatment, there is no need to use laboratory animals. This contributes to a reduction in laboratory animals in line with the 3R approach in biomedical research based on the adopted Directive 2010/63/EU, which sets the full replacement of experimental animals for scientific purposes as an ultimate goal.

Another limitation is the use of unsorted MSCs for the in vitro experiments, resulting in a heterogeneous cell population with a higher variation for all outcome parameters. Unfortunately, to the authors knowledge, there is no established marker to select canine MPCs from the synovium. However, this study provides some insights in the suitability of known markers for further investigations.

### Impact and future directions

The observations of this study impact the application of SM-MSCs in cell-based treatments of OA in multiple ways. Firstly, the use of autologous SM-MSCs from the OA joint should be critically considered, as their regenerative capacity is inferior to normal SM-MSCs. However, to reach final conclusions regarding their regenerative capacity, the behaviour of both normal and OA SM-MSCs should be investigated in an in vivo situation. Secondly, in case of exogenous MSC implantation, the OA environment may inhibit the regenerative capacity of the implanted MSCs. Considering that the effects of the OA environment are still present on cSM-MSCs in vitro at least until passage 2*,* the in vitro culture of these cells could serve as model to develop new methods and test the developed strategies in improving the performance of MSCs as a cell-based treatment strategy.

## Conclusions

To exploit the regenerative capacity of synovial membrane-derived progenitor cells, more in-depth knowledge is needed about their role in the normal joint homeostasis and OA. This study showed that the OA environment has a negative effect on the regenerative capacity of cSM-MSCs indicated by the decreased CFU and population doublings, and decreased chondrogenic but enhanced osteogenic potency compared to normal cSM-MSCs. Furthermore, in the OA synovial membrane the MSC/MPC markers CD90, CD44, CD73, and CD271 were decreased, indicating a loss of MSC/MPC phenotype or a depletion of progenitor cells in the synovial membrane.

## Supplementary Information

**Additional file 1**. Supplementary figures and tables.

## Data Availability

All data generated or analysed during this study are included in this published article in the form of graphs. Additional datasets used and/or analysed during the current study are available from the corresponding author on reasonable request.

## Abbreviations

MPC: Mesenchymal progenitor cell
MSC: Mesenchymal stem/stromal cell
SM: Synovial membrane
OA: Osteoarthritis
RT-qPCR: Reverse transcriptase quantitative polymerase chain reaction
HE: Haematoxylin Eosin
OARSI: Osteoarthritis Research Society International
FBS: Foetal bovine serum
CFU: Colony-forming unit
AsAP: Ascorbic acid 2-phosphate
bFGF: Basic fibroblast growth factor
P: Passage
PD: Population doublings
NBF: Neutral buffered formalin
GAG: Glycosaminoglycan
DMMB: Dimethyl methylene blue
RT: Room temperature
FSC: Forwards scatter
SSC: Side scatter
ROI: Region of interest
ES: Effect size
HG: Hedge’s G (effect size)
CD: Cliff’s delta (effect size)
ISCT: International Society for Cellular Therapy
COL: Collagen
