# Supplementary material for: Synovial membrane-derived mesenchymal progenitor cells from osteoarthritic joints in dogs possess lower chondrogenic-, and higher osteogenic capacity compared to normal joints
Source: Stem Cell Res Ther. 2022 Sep 5;13:457. doi: 10.1186/s13287-022-03144-z (PMC9446738; doi:10.1186/s13287-022-03144-z)
Supplement: Supplementary file 1 — Additional file 1. Supplementary figures and tables. [file 13287_2022_3144_MOESM1_ESM.docx]

**Supplementary Material**


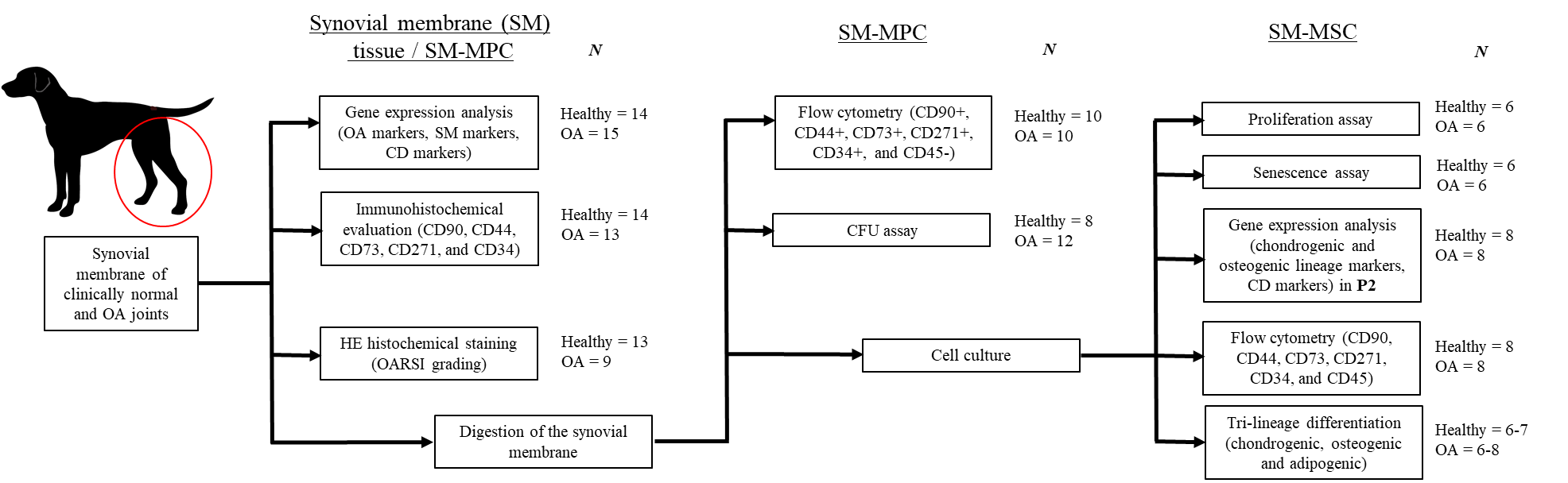


**Supplementary figure 1: Flow-chart of the used techniques.** OA: Osteoarthritis; CFU: Colony forming unit; P2: Passage 2; N: the number of donors used


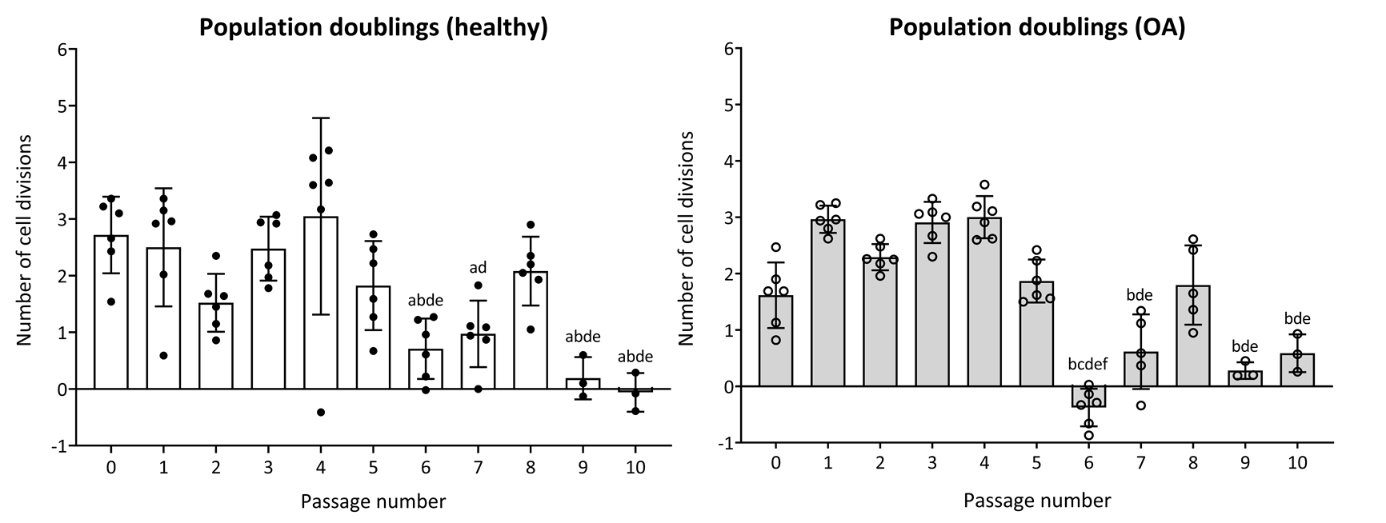


**Supplementary figure 2:** Population doublings of normal (A) and osteoarthritic (OA) (B) synovial membrane-derived MSC per passage (P). Data are presented as the mean ± standard deviation. Dots represent individual donors. Letter symbols indicate significant differences (p < 0.05) between passages (a: compared to P0; b: compared to P1; c: compared to P2; d: compared to P3; e: compared to P4; f: compared to P5) .


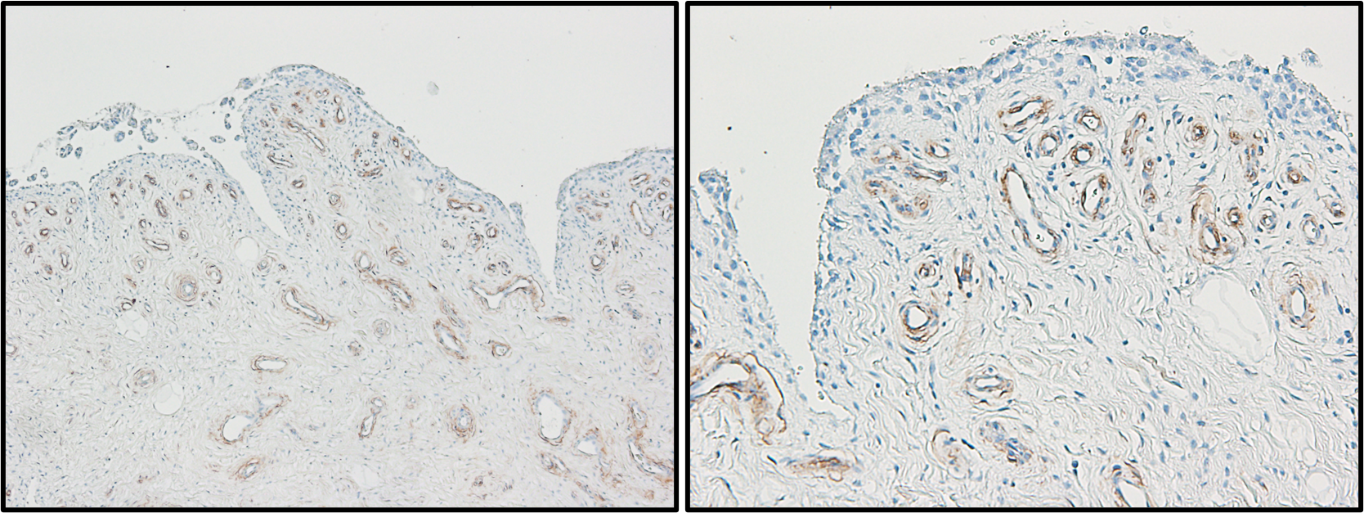


**Supplementary figure 3:** CD90 expression in human osteoarthritic synovial membrane. The same antibody and immunohistochemical staining protocol was used as for the canine synovial membrane. Marginal expression was seen in the perivascular region. 4x and 10x objectification

**Supplementary table 1: Donor information.** Joint status; clinically normal (healthy) or osteoarthritic (OA); Donor number (no.); the breed of the dog; the age in months; the body weight in kg; and the sex and if the animal was neutered. Application: the parameter that was investigated for this donor; F (Flow cytometry), C (Cell culture), P (RT-qPCR), H (Histological evaluation (OARSI scoring or immunohistochemistry).

| **Joint status** | **Donor No.** | **Breed** | **Age (months)** | **Body weight (kg)** | **Sex** | **Application** |
| --- | --- | --- | --- | --- | --- | --- |
| **Healthy** | 1 | Crossbreed | Unknown | 25 | Male | F |
|  | 2 | Crossbreed | Unknown | 25 | Male | F |
|  | 3 | Crossbreed | 16 | 25 | Male | F, C |
|  | 4 | Labrador Retriever | 7 | 25 | Male | H, P, C |
|  | 5 | Crossbreed | 14 | 25 | Male | H, F, P, C |
|  | 6 | Crossbreed | 14 | 25 | Male | F, C |
|  | 7 | Crossbreed | 15 | 25 | Female | F, P, C |
|  | 8 | Crossbreed | 15 | 25 | Female | F, P, C |
|  | 9 | Crossbreed | 21 | 26 | Male | F, C |
|  | 10 | Crossbreed | 20 | 26 | Male | H |
|  | 11 | Crossbreed | 15 | 24 | Female | H, P |
|  | 12 | Crossbreed | 15 | 24 | Female | H, P |
|  | 13 | Crossbreed | 15 | 24 | Female | H, P |
|  | 14 | Crossbreed | 31 | 28 | Female | H, F |
|  | 15 | Crossbreed | 31 | 25 | Female | H |
|  | 16 | Crossbreed | 30 | 23 | Female | F, P |
|  | 17 | Crossbreed | 15 | 24 | Female | H |
|  | 18 | Crossbreed | 35 | 25 | Female | H |
|  | 19 | Crossbreed | 18 | 26 | Female | H, P |
|  | 20 | Crossbreed | 11 | 24 | Male | H |
|  | 21 | Crossbreed | 17 | 26 | Male | H |
|  | 22 | Crossbreed | 22 | 20 | Female | H |
|  | 23 | Crossbreed | Unknown | Unknown | Unknown | F |
|  | 24 | Crossbreed | 18 | 26 | Female | H, P |
|  | 25 | Crossbreed | 8 | 25 | Male | H |
|  | 26 | Crossbreed | 15 | 25 | Female | P |
|  | 27 | Crossbreed | Unknown | Unknown | Unknown | H |
|  | 28 | Crossbreed | 15 | 22 | Male | H, P |
|  | 29 | Crossbreed | 16 | 27 | Male | F, P |
|  | 1 | Crossbreed | 51 | 50 | Female | F, P, C |
|  | 2 | American Staffordshire Terrier | 59 | 33 | Male | H, F, P, C |
| **OA** | 3 | Boerboel | 25 | 61 | Female | H, F, P, C |
|  | 4 | Labrador Retriever | 75 | 32 | Female (neutered) | H |
|  | 5 | Golden Retriever | 58 | 32 | Female (neutered) | H, F |
|  | 6 | American Bulldog | 42 | 48 | Male | F, C |
|  | 7 | Mastín Español | 16 | 65 | Female | F, P, C |
|  | 8 | Crossbreed | 50 | 37 | Male (neutered) | F, P, C |
|  | 9 | Crossbreed | 27 | 54 | Male (neutered) | H, F |
|  | 10 | Labrador crossbreed | 20 | 34 | Male | H, F, C |
|  | 11 | Labrador crossbreed | 80 | 32 | Male | H |
|  | 12 | Labrador Retriever | 66 | 35 | Male (neutered) | H |
|  | 13 | Labrador Retriever | 12 | 32 | Male | P |
|  | 14 | Labrador Retriever | 168 | 31 | Female (neutered) | H, P |
|  | 15 | American Bulldog | 59 | 29 | Male (neutered) | H |
|  | 16 | American Staffordshire Terrier | 68 | 38 | Male | H, P |
|  | 17 | Golden Retriever | 41 | 30 | Male (neutered) | F, C |
|  | 18 | Bullmastiff | 40 | 46 | Female (neutered) | H, P, C |
|  | 19 | Labrador Retriever | 139 | 31 | Male (neutered) | H, F, P, C |
|  | 20 | Labrador Retriever | 55 | 30 | Female (neutered) | F, P, C |
|  | 21 | Siberian Husky | 130 | 30 | Male | F, P, C |
|  | 22 | Border Collie | 39 | 17 | Male | H, F, P, C |

**Supplementary table 2: Immunohistochemical staining protocols.** No.: Number; Ig: Immunoglobulin; min: minutes; RT: room temperature; PBS: phosphate-buffered saline; BSA: Bovine serum albumin

| **Target** | **Catalog No.** | **Manufacturer** | **Host** | **Antibody Ig fraction** | **Antigen Retrieval** | **Block** | **Dilution 1^st^ Antibody** | **Secondary Antibody** | **Detection system** | **Isotype control** |
| --- | --- | --- | --- | --- | --- | --- | --- | --- | --- | --- |
| **CD90** | Ab92574 | Abcam | Rabbit | Mab IgG | No antigen retrieval | 1st: Dual Endogenous Enzyme Block (DAKO,S2003), RT, 10 min | 1:750 in PBS/BSA 5% | EnVision K4003 | DAB peroxidase substrate solution (DAKO, K3468) | DAKO X0903 |
|  |  |  |  |  |  | 2nd: PBS/BSA 5%, RT, 30 min |  |  |  |  |
| **CD73** | LS-B8284 | IHCplus | Rabbit | Pab IgG | No antigen retrieval | 1st: Dual Endogenous Enzyme Block (DAKO,S2003), RT, 10 min | 1:1000 in PBS/BSA 5% | EnVision K4003 | DAB peroxidase substrate solution (DAKO, K3468) | DAKO X0931 |
|  |  |  |  |  |  | 2nd: PBS/BSA 5%, RT, 30 min |  |  |  |  |
| **CD44** | MA1-10225 | Thermo Fisher | Rat | Mab IgG2b | No antigen retrieval | 1st: Dual Endogenous Enzyme Block (DAKO,S2003), RT, 10 min | 1:2000 in PBS/BSA 5% | Chemicon AP136P | DAB peroxidase substrate solution (DAKO, K3468) | SouthernBiotech, 0118-01 |
|  |  |  |  |  |  | 2nd: PBS/BSA 5%, RT, 30 min |  |  |  |  |
| **CD271** | 14-9400-82 | eBioscience | Mouse | Mab IgG1-κ | No antigen retrieval | 1st: Dual Endogenous Enzyme Block (DAKO,S2003), RT, 10 min | 1:500 in PBS/BSA 5% | EnVision K4001 | DAB peroxidase substrate solution (DAKO, K3468) | DAKO X0931 |
|  |  |  |  |  |  | 2nd: PBS/BSA 5%, RT, 30 min |  |  |  |  |
| **CD34** | bs-0646R | MyBiosource | Mouse | Mab IgG1 | Citrate buffer; 98 °C, 20 min | 1st: Dual Endogenous Enzyme Block (DAKO,S2003), RT, 10 min | 1:1500 in PBS/BSA 5% | EnVision K4001 | DAB peroxidase substrate solution (DAKO, K3468) | DAKO X0931 |
|  |  |  |  |  |  | 2nd: PBS/BSA 5%, RT, 30 min |  |  |  |  |
| **Collagen type I** | Ab6308 | Abcam | Mouse | Mab IgG1 | 1st : Pronase; 37 °C, 30 min | 1st: Dual Endogenous Enzyme Block (DAKO,S2003), RT, 10 min | 1:1500 in PBS/BSA 5% | EnVision K4001 | DAB peroxidase substrate solution (DAKO, K3468) | DAKO X0931 |
|  |  |  |  |  | 2nd: Hyaluronidase; 37 °C, 30 min | 2nd: PBS/BSA 5%, RT, 30 min |  |  |  |  |
| **Collagen type II** | II-II6B3 | DSHB | Mouse | Mab IgG1 | 1st : Pronase; 37 °C, 30 min | 1st: Dual Endogenous Enzyme Block (DAKO,S2003), RT, 10 min | 1:2000 in PBS/BSA 5% | EnVision K4001 | DAB peroxidase substrate solution (DAKO, K3468) | DAKO X0931 |
|  |  |  |  |  | 2nd: Hyaluronidase; 37 °C, 30 min | 2nd: PBS/BSA 5%, RT, 30 min |  |  |  |  |
